# Contribution of protein Gar1 to the RNA-guided and RNA-independent rRNA:Ψ-synthase activities of the archaeal Cbf5 protein

**DOI:** 10.1038/s41598-018-32164-0

**Published:** 2018-09-14

**Authors:** Ryosuke Fujikane, Isabelle Behm-Ansmant, Anne-Sophie Tillault, Christine Loegler, Valérie Igel-Bourguignon, Evelyne Marguet, Patrick Forterre, Christiane Branlant, Yuri Motorin, Bruno Charpentier

**Affiliations:** 1 Université de Lorraine, CNRS, UMR 7365 Ingénierie Moléculaire et Physiopathologie Articulaire (IMoPA), F-54500 Nancy, France; 2 Institute for Integrative Biology of the Cell (I2BC), CEA, CNRS, Université Paris-Sud, Université Paris-Saclay, F-91198 Gif-sur-Yvette cedex, France; 3 Université de Lorraine, CNRS, INSERM, UMS2008 IBSLor, F-54500 Nancy, France; 4 Present Address: Fukuoka Dental College, Department of Physiological Sciences and Molecular Biology, Section of Cellular and Molecular Regulation, 2-15-1 Tamura, Sawara-ku, Fukuoka 814-0193 Japan; 5 Present Address: Department of Biological Sciences, University of Lethbridge, Lethbridge, Alberta Canada; 6 Institut Pasteur, Département de Microbiologie, 25 rue du Dr Roux F-7505 Paris, France

## Abstract

Archaeal RNA:pseudouridine-synthase (PUS) Cbf5 in complex with proteins L7Ae, Nop10 and Gar1, and guide box H/ACA sRNAs forms ribonucleoprotein (RNP) catalysts that insure the conversion of uridines into pseudouridines (Ψs) in ribosomal RNAs (rRNAs). Nonetheless, in the absence of guide RNA, Cbf5 catalyzes the *in vitro* formation of Ψ_2603_ in *Pyrococcus abyssi* 23S rRNA and of Ψ_55_ in tRNAs. Using gene-disrupted strains of the hyperthermophilic archaeon *Thermococcus kodakarensis*, we studied the *in vivo* contribution of proteins Nop10 and Gar1 to the dual RNA guide-dependent and RNA-independent activities of Cbf5 on 23S rRNA. The single-null mutants of the *cbf5*, *nop10*, and *gar1* genes are viable, but display a thermosensitive slow growth phenotype. We also generated a single-null mutant of the gene encoding Pus10, which has redundant activity with Cbf5 for *in vitro* formation of Ψ_55_ in tRNA. Analysis of the presence of Ψs within the rRNA peptidyl transferase center (PTC) of the mutants demonstrated that Cbf5 but not Pus10 is required for rRNA modification. Our data reveal that, in contrast to Nop10, Gar1 is crucial for *in vivo* and *in vitro* RNA guide-independent formation of Ψ_2607_ (Ψ_2603_ in *P*. *abyssi*) by Cbf5. Furthermore, our data indicate that pseudouridylation at orphan position 2589 (2585 in *P*. *abyssi*), for which no PUS or guide sRNA has been identified so far, relies on RNA- and Gar1-dependent activity of Cbf5.

## Introduction

Pseudouridine (Ψ) is the most abundant RNA modification (for review^1–3^). This modified nucleoside is generated by site-specific C5-ribosyl isomerization of uridine (U) in pre-existing RNA transcripts. Formation of Ψ in RNAs can increase base stacking^4^ and rigidify the sugar-phosphate backbone^1,5,6^. More specifically, Ψ can stabilize RNA 3D conformation by water bridge formation, as demonstrated in tRNAs^7,8^. A cluster of Ψs can modulate the conformation of a large RNA domain as shown for helix 69 – H69 – in rRNA^9^. It is now firmly established that some evolutionarily conserved Ψs in tRNAs and rRNAs are required for accurate function of these non-coding RNAs (ncRNAs) in translation (for review^2^, and^10–20^). Similarly, some of the Ψs in U-rich small nuclear RNAs (UsnRNAs) are important for pre-mRNA splicing^2,21,22^. Moreover, recent transcriptome-wide maps of pseudouridylation detected a large number of Ψs in eukaryotic mRNAs^23,24^ but their precise function is not yet firmly established.

The conversion of uridine (U) into Ψ is catalyzed by specific RNA:Ψ-synthases (PUS) (for review^25^). Five families of PUS have been defined based on protein sequence conservation with each of the bacterial TruA, TruB, TruD, RluA, and RsuA proteins (for review^25^). Comparative analysis of eukaryotic genomes revealed the presence of members of the same five families of PUS in Eukarya. Studies on archaea identified a rather limited number of putative genes encoding PUS, while in addition to PUS homologous to bacterial PUS, a sixth family of PUS was detected in eukaryotes. This sixth family of PUS has an archaeal representative – Pus10 of the archaeon *Pyrococcus abyssi*^26,27^.

In archaea and eukaryotes, the catalysts for Ψ synthesis are either stand-alone PUS proteins or ribonucleoprotein particles with PUS activities that are referred to as box H/ACA small RNPs (sRNPs) in archaea and as box H/ACA small nucleolar RNPs (snoRNPs) in eukaryotes (for review^25,28,29^). In archaea, box H/ACA sRNPs are formed by association of distinct H/ACA sRNAs with a constant set of four proteins including the TruB-like PUS aCBF5, the ribosomal protein L7Ae, and proteins aNOP10 and aGAR1 (thereafter named Cbf5, L7Ae, Nop10, and Gar1, respectively)^30,31^. In absence of sRNA, protein complexes can be formed in which Nop10 and Gar1 interact independently with the catalytic domain of Cbf5^32–34^. The H/ACA sRNAs act as guide RNAs ensuring the specificity of the RNP enzyme. Each H/ACA sRNA contains at least one hairpin-tail structure with a large internal loop, which forms base-pair interactions with a target RNA sequence. Two duplexes are then formed on each side of the unpaired U to be modified by Cbf5. Cbf5 interacts together with the box H/ACA sRNA and proteins Nop10 and Gar1^31,32,35–37^. The Gar1 protein is dispensable for total modification of substrate RNAs in single-turnover reactions^31^, but it enhances the kinetics of the reaction^30,31,38,39^. By modulating the formation of open and closed states of the catalytic center, Gar1 facilitates substrate RNA loading, its efficient placement and the release of the modified RNA^38,40–42^.

The 2′-*O*-methylations and U to Ψ conversions in RNAs have been suggested to play a role in the capacity of archaea to grow at high temperatures. Current knowledge of RNA modification in archaea showing a relatively low number of PUS encoding genes compared to other organisms raises the question of the enzymatic mechanism required for the generation of the rather large number of Ψs present in RNAs of this kingdom. The use of both classical protein catalysts and H/ACA sRNP catalysts and the multi-functionality and wide specificity of PUS in archaea^43^ may limit the number of enzymes involved in archaeal RNA modification. However, as some of the information in this field was obtained through *in vitro* assays, complementary *in vivo* experiments are needed to elucidate this puzzling question. For instance, Cbf5, which is the only representative of the TruB family in archaea and acts as the catalytic subunit of the H/ACA sRNPs^30,31^, may also function as a stand-alone enzyme. *In vitro* assays using the *P*. *abyssi* Cbf5 showed that it is able to catalyze Ψ_55_ formation in the loop of the TΨC arm of elongator tRNAs^26,44–49^. One argument against this hypothesis is the finding that Cbf5 is not essential for the generation of this modification *in vivo* in *Haloferax volcanii*, in which this activity is carried out by the Pus10 PUS^26,50–54^. Nevertheless, the possibility that the tRNA:Ψ_55_-synthase activity of Cbf5 is used in other archaeal species cannot be excluded. Furthermore, Cbf5 may have a stand-alone activity in modification of rRNA. Indeed, despite of a deep computer analysis, we failed to identify any H/ACA sRNA able to guide formation of the two Ψs respectively detected at positions 2585 and 2603 in the *P*. *abyssi* in 23S rRNA^55^, and we found that recombinant Cbf5 in presence of proteins Nop10 and Gar1 can modify *in vitro* residue U_2603_ in a transcribed 23S rRNA fragment that can fold into a stem-loop structure showing some similarity with the TΨC arm of tRNAs^48,55^. As this TΨC-like-loop structure has not been predicted in classical 23S rRNA folding models, the possible RNA-independent activity of Cbf5 in archaeal 23S rRNA modification still remains to be demonstrated *in vivo*. Of note, the previous data did not reveal any possible activity of either Cbf5 alone or of the Cbf5–Nop10–Gar1 protein complex at position U_2585_, raising the question of the catalyst used at this position^55^.

Here, we describe successful isolation of *T*. *kodakarensis* single knockout mutants Δ*cbf5*, Δ*nop10*, and Δ*gar1* of the ORFs encoding the Cbf5, Nop10, and Gar1 components of H/ACA sRNPs, as well as a Pus10 single knockout mutant Δ*pus10*. Obtaining these mutants indicates that each of these proteins taken separately is not essential for viability. However, growth of *T*. *kodakarensis* at high temperatures was markedly slowed down. The null mutants were used to identify the catalysts responsible for *in vivo* pseudouridylation at the orphan positions U_2589_ (U_2585_ in *P*. *abyssi*) and U_2607_ (U_2585_ in *P*. *abyssi*) in 23S rRNA. Altogether, our data reveal a general function for Gar1 in the RNA guide-dependent and RNA-independent activities of Cbf5.

## Results

### Construction of RNA:Ψ-synthases and H/ACA RNP components gene deletion mutants

**Figure 1:**
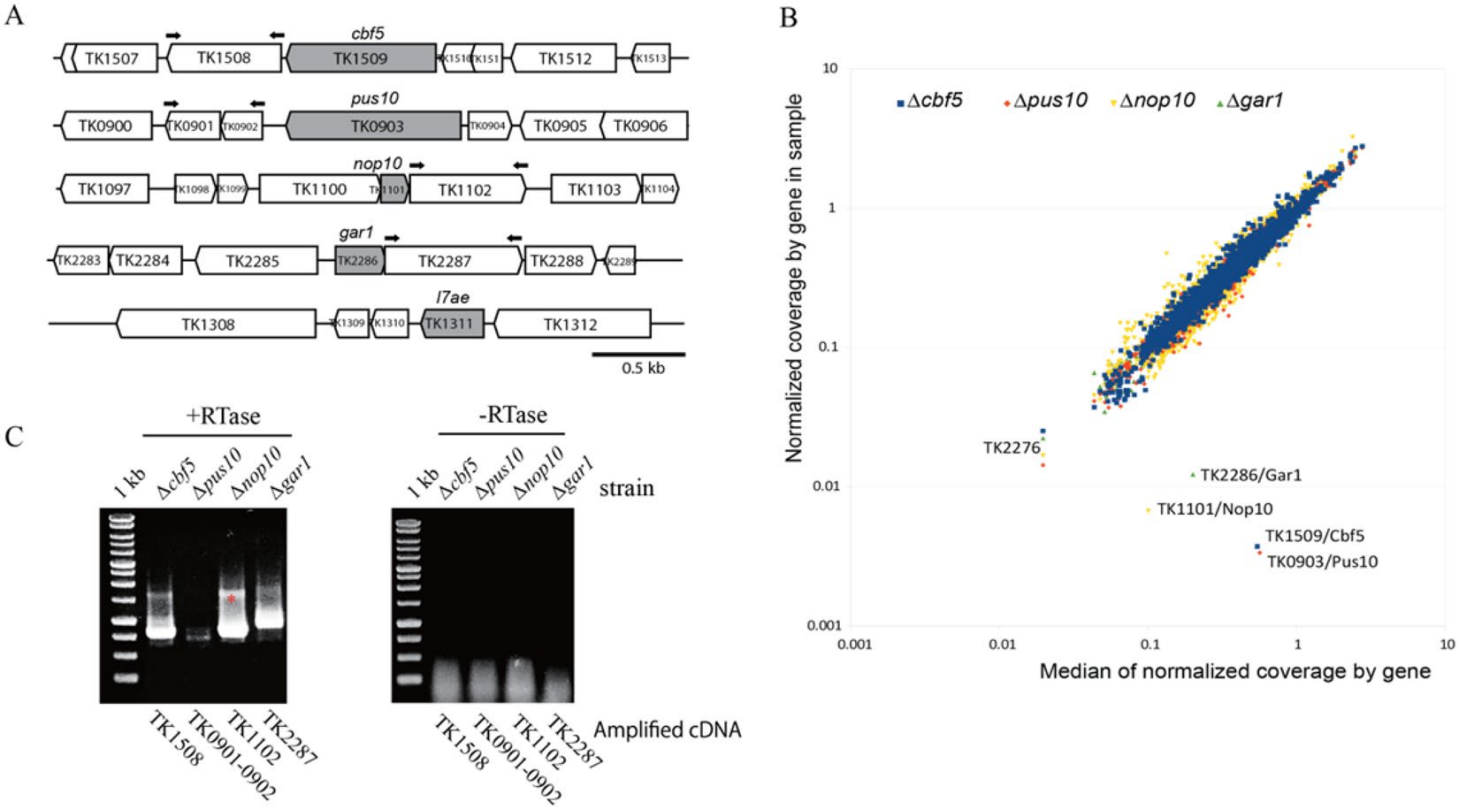
Construction of disruption mutants. (**A**) Schematic diagram of the loci targeted for disruption. The target genes are in gray. The black arrows indicate the primers for RT-PCR analysis (panel C and Table S3). (**B**) Normalized reads’ coverage as a function of the median coverage observed for all four deleted *T*. *kodakarensis* strains. Colors correspond to the coverage observed for ΔTK1509/*cbf5* (blue), ΔTK0903/*pus10* (red), ΔTK1101/*nop10* (yellow) and ΔTK2286/*gar1* (green) strains. Identity of outlier points is indicated. TK2276 (*pyrF*) is affected in all four strains analyzed. (**C**) Disruption of each targeted ORFs does not impair transcription of their respective downstream contiguous ORF. RT-PCR reactions (gel + RTase) were performed with oligonucleotide primers (Table S3) complementary to the coding sequence of the target gene’s adjacent ORFs (indicated below each lane). The RT-PCR products were fractionated by 0.8% agarose gel electrophoresis. The bands correspond to RNA amplification since no amplified products were obtained in a reaction mixture lacking reverse transcriptase (-RTase). The asterisk indicates non-specific amplification.

We performed sequence analysis of the *T*. *kodakarensis* genome^56^ in the aim of identifying ORFs for the PUS Cbf5 and Pus10, and for protein components L7Ae, Nop10, and Gar1 of the H/ACA RNPs. The protein-protein BLAST program using the amino acid sequences of *P*. *abyssi* PUS and H/ACA sRNP protein components as queries identified a single ortholog of Cbf5 (TK1509), Pus10 (TK0903), L7Ae (TK1311), Nop10 (TK1101), and Gar1 (TK2286) in *T*. *kodakarensis* genome (Fig. 1A and Supplementary data, Tables S1 and S2). To analyze how those proteins contribute to pseudouridylation of RNAs *in vivo*, we disrupted their genes using the gene targeting method (Supplementary data, Fig. S1)^57,58^. The gene disruption vectors harboring the selectable marker *pyrF* (TK2276), which encodes orotidine-5′-monophosphate decarboxylase, and 1 kb of the 5′ and 3′ flanking regions of each target gene were constructed as described in Methods. They were used to transform the *T*. *kodakarensis* strain KU216 showing uracil auxotrophy due to a non-functional *pyrF* locus^59^. Transformants harboring integration of the plasmid into the chromosome through homologous recombination were selected from the KU216 strain for uracil autotrophy. The intermediary *pyrF*^+^ strains were then selected by 5-FOA resistance to obtain gene knockout mutant strains^58^. Using this strategy, we obtained colonies for the single knockout mutants Δ*cbf5*, Δ*pus10*, Δ*nop10*, and Δ*gar1*, indicating that, taken separately, none of these genes were essential for cell viability. However, this was not the case for the ORF encoding the ribosomal protein L7Ae, for which we did not succeed in isolating a viable disrupted mutant.

In order to validate the deletion of desired ORFs in mutant *T*. *kodakarensis* strains and to exclude potential off-target integration of *pyrF* gene used in knockout strategy, we performed whole genome sequencing and analysis of generated *T*. *kodakarensis* mutants. Fragmented genomic DNA extracted from each mutant was converted to the library and sequenced on Illumina HiSeq1000. Sequencing reads (~0.6–1 mln/library, 20–30x mean coverage) were aligned to the reference genome and coverage was calculated by each *T*. *kodakarensis* ORF. Figure 1B shows the normalized gene coverage versus the median value for all four sequenced strains. As clearly shown on the graph, only a single point for each strain demonstrates strong deviation from the median, those correspond to the expected ORFs deleted in the respective strain. As anticipated, ORF TK2276 (alias gene *pyrF*) is absent in all four strains used. More detailed information on the deletions is given in Supplementary data (% of covered positions by gene and sequencing reads aligned in the regions of expected deletions in Fig. S2). Altogether these data unambiguously confirm that only one ORF in each *T*. *kodakarensis* mutant was affected by deletion and no undesired off-target integration/popping out of *pyrF* gene occurred.

Since in archaeal genomes, many genes are organized in clusters or in operons^60^, it was possible that the individual ORF disruption affected the structure of the operon or of the cluster. As a consequence, the transcription of downstream ORFs or genes may also be affected. To check for this possibility, we isolated total RNAs from the null mutant cells and performed RT-PCR to detect mRNA produced from the downstream gene. Bands corresponding to transcribed products from the downstream genes were detected (Fig. 1C), indicating that the gene deletions did not affect transcription of the downstream genes. Hence, the observed phenotype for each null mutant was expected to be only related to the lack of the disrupted ORF function.

### Growth phenotype of the deletion mutant strains

**Figure 2:**
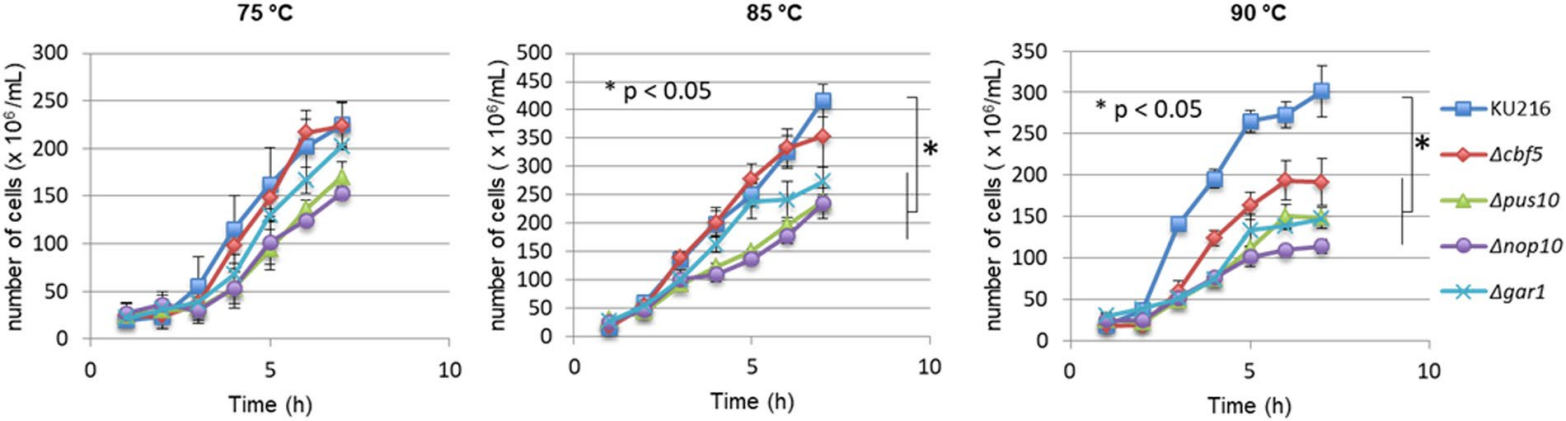
Analysis of the effect of gene disruption on cell growth. The various *T*. *kodakarensis* wild type (KU216) and mutant strains were grown in ASW-YT medium. Cultures were carried out at the optimal growth temperature of 85 °C, or at 75 °C and 90 °C, and cell growth was monitored by counting the number of cells with Thoma’s cell counter during the course of the culture. The mean values obtained from three independent experiments and the standard deviations are shown with bars. Significance values of *p < 0.05 relative to KU216. square: KU216, diamond: Δ*cbf5*, triangle: Δ*pus10*, circle: Δ*nop10*, and cross: Δ*gar1*.

The isolated mutant strains were cultivated at 85 °C, which is the optimal growth temperature for *T*. *kodakarensis*^61^, as well as at 75 °C and 90 °C. The number of growing cells as a function of time was counted with Thoma’s cell counter under an optical microscope (Fig. 2). At 75 °C and at 85 °C, the mutant strains Δ*pus10*, Δ*nop10*, and Δ*gar1* showed visible growth retardation compared to the wild type strain and the final yields of cells were only three-fourths for Δ*gar1* and two-thirds for Δ*pus10* and Δ*nop10*. Remarkably, in Δ*pus10* and Δ*nop10* strains, cellular aggregation was observed after 4 hours cultivation (Supplementary data, Fig. S3). Aggregation of archaeal cells has already been observed in *Sulfolobus* after UV irradiation^62,63^, and in *Halobacterium* after heat shock or divalent ion treatment^64^. This may suggest that the disruption of *nop10* or *pus10* genes causes some cellular stress leading to cellular aggregation. As proteins Nop10 and Gar1 are known to contribute to Cbf5 activity and as no apparent phenotype was associated with disruption of the *cbf5* gene at suboptimal and optimal growth temperatures, one possible explanation is that Nop10 and Gar1 have additional cellular functions that are not connected with RNA modification.

All the mutant strains showed significant growth defects at 90 °C. These data suggest that pseudouridines synthesized by Cbf5, Pus10, and the H/ACA sRNPs contribute to rRNA and/or tRNA stabilization at high temperatures and/or are even important for ribosome biogenesis and activity.

### Cbf5 but not Pus10 is required for Ψ2589(2585) and Ψ2607(2603) formation in 23S rRNA

**Figure 3:**
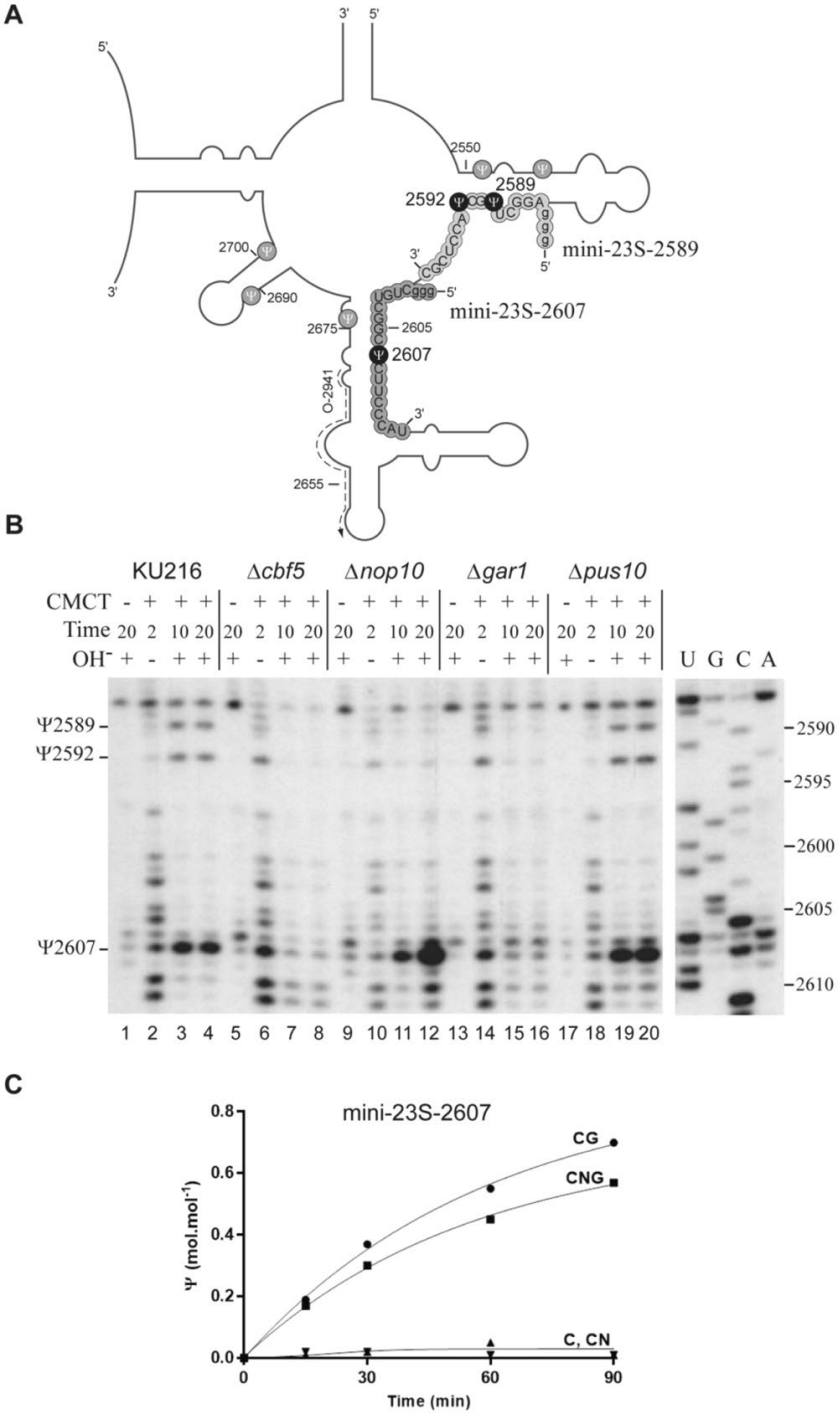
The effect of gene disruption on Ψ formation in the domain V of *T*. *kodakarensis* 23S rRNA. (**A**) Ψs identified in this work in the *T*. *kodakarensis* 23S rRNA, which are conserved in the *P*. *abyssi* 23S rRNA are indicated by black dots. The grey dots correspond to other Ψs detected in *P*. *abyssi* 23S rRNA in a previous work^55^. The secondary structure of the *T*. *kodakarensis* 23S rRNA is adapted from Piekna *et al*.^16^. The sequence of the two substrates mini–23S–2607 and mini–23S–2589, synthesized by *in vitro* transcription and used *in vitro* in Panel C (mini–23S–2607) and in Fig. 5 (mini–23S–2589), is highlighted. (**B**) Identification of Ψs in the *T*. *kodakarensis* 23S rRNA by the CMCT-RT method. As indicated at the top of each lane, total RNAs extracted from the various *T*. *kodakarensis* strains were treated in the absence (−) or the presence of CMCT (+), for 2, 10, and 20 min as described in Methods. The CMCT treatment was (+) or was not (−) followed by an alkaline treatment at pH 10.4. The positions of Ψs were identified by primer extension analysis using oligonucleotide O-2941 (represented by the dashed arrow) as described in Methods. Digital image of the autoradiogram was obtained by scanning the x-ray film. The presence of Ψs is revealed by the appearance of alkali-resistant RT stops. Lanes U, G, C, and A correspond to the RNA sequencing ladder. The positions of nucleotides in 23S rRNAs are indicated to the left of the autoradiogram. The two panels correspond to a cropping of two sections of the same autoradiogram. The full-length gel is presented in Supplementary Figure S4. (**C**) Time course analysis of *in vitro* modification by recombinant proteins in RNA substrate mini–23S–2607. The RNA substrate was radiolabeled by the incorporation of [α-^32^P]CTP during *in vitro* transcription. The RNA was incubated with different protein combinations: Cbf5 alone (**C**), Cbf5 and Nop10 (CN), Cbf5, and Gar1 (CG), and a set of the three proteins (CNG). At each time point, an aliquot of the reaction mixture was analyzed by 2D-TLC. The radioactivity was quantified by PhosphorImager analysis. The quantities of Ψ nucleotides formed are expressed in moles per mole of mini–23S–2607.

We next conducted phenotypic analysis of the null mutants by looking for the presence of Ψs in 23S rRNA using the CMCT-RT approach. We focused this more extensive analysis on the Domain V region of 23S archaeal rRNA spanning residues 2588 to 2612 (Fig. 3A). This region contains three Ψ residues at positions 2589, 2592, and 2607. Pseudouridine at position 2607 (2603 in *P*. *abyssi*) can be formed in a RNA guide-independent way^55^, while Ψ_2589_ (Ψ_2585_ in *P*. *abyssi*) still have no associated RNA guide and complex Cbf5–Nop10–Gar1 is not capable of forming Ψ_2585_ *in vitro*^55^. RNA guide-dependent residue Ψ_2592_ (Ψ_2588_ in *P*. *abyssi*) was used as control.

Total RNAs extracted from the various strains were treated with CMCT as described in Methods and alkaline resistant Ψ–CMC adducts were detected in the region analyzed by primer extension analysis, at the expected positions: nucleotides U_2589_, U_2592_, and U_2607_ (corresponding to U_2585_, U_2588_, and U_2603_ in *P*. *abyssi*) (Fig. 3A,B, lanes 3 and 4). This analysis complements our previous study on *P*. *abyssi* 23S rRNA^55^, and reveals the presence of a highly conserved modification at these positions in both *T*. *kodakarensis* and *P*. *abyssi* 23S rRNAs. None of the three modifications occurred in the absence of Cbf5 whereas all of them were present in the *pus10*-null strain (Fig. 3B, compare lanes 7 and 8 with lanes 19 and 20). These data demonstrated that Cbf5 and not Pus10 activity is required *in vivo* for Ψ formation at these three positions.

### Nop10 is dispensable but Gar1 is crucial for Ψ2607(2603) formation in 23S rRNA

The *P*. *abyssi* heterotrimer complex Cbf5–Nop10–Gar1 was previously found to modify *in vitro* residue U_2603_ when it is contained in an RNA substrate composed of a 22-nucleotide long fragment of 23S rRNA^55^. Surprisingly, in the present *in vivo* study, we observed that the disruption of ORFs *nop10* or *gar1* in *T*. *kodakarensis* had a different effect on 23S rRNA modification at the corresponding position U_2607_. The modification Ψ_2607_ was still detected in mutant Δ*nop10*, but was no longer detected in mutant Δ*gar1* (Fig. 3B, compare lane 12 with lane 16). These data show that Ψ_2607_ formation *in vivo* requires the presence of Gar1 but does not require the presence of Nop10. In contrast, both Ψ_2589_ and Ψ_2592_ were absent in all three strains with deletions of *cbf5*, *nop10*, or *gar1* genes (Fig. 3B).

In a previous work, we showed that the *P*. *abyssi* Pab40 H/ACA sRNA guides modification of 23S rRNA at position U_2588_ *in vitro*^55^. Modification of the equivalent nucleotide U_2592_ in *T*. *kodakarensis* 23S rRNA is likely to be also guided by an H/ACA sRNA since the gene sequence for a Pab40 homolog (Tko1) is present in the *T*. *kodakarensis* genome^55^. The absence of Nop10 or Gar1 has the same effect as the absence of Cbf5, since it completely stopped the formation of Ψ_2592_ in 23S rRNA (Fig. 3B, lanes 11 and 12, and lanes 15 and16). These data are in agreement with the implication of proteins Nop10 and Gar1 in the activity of a conserved H/ACA RNP catalyzing Ψ_2592(2588)_ formation.

To further pursue our investigation of the role of the auxiliary proteins, we compared single-turnover activities of the free Cbf5 protein, and the Cbf5–Nop10 (CN), Cbf5–Gar1 (CG), and Cbf5–Nop10–Gar1 (CNG) protein mixtures (Fig. 3C), for *in vitro* modification of an excess of the 23S rRNA fragment mini–23S–2607 (Fig. 3A). Formation of Ψ_2607_ was observed upon incubation of the labeled mini–23S–2607 RNA with Cbf5–Gar1 and Cbf5–Nop10–Gar1, but not with Cbf5 alone or in association with Nop10. These *in vitro* data confirm that Gar1, but not Nop10, is required for the non-RNA-guided activity of Cbf5 at position 2607(2603) in 23S rRNA.

### Contribution of Gar1 to H/ACA RNP enzyme activity

**Figure 4:**
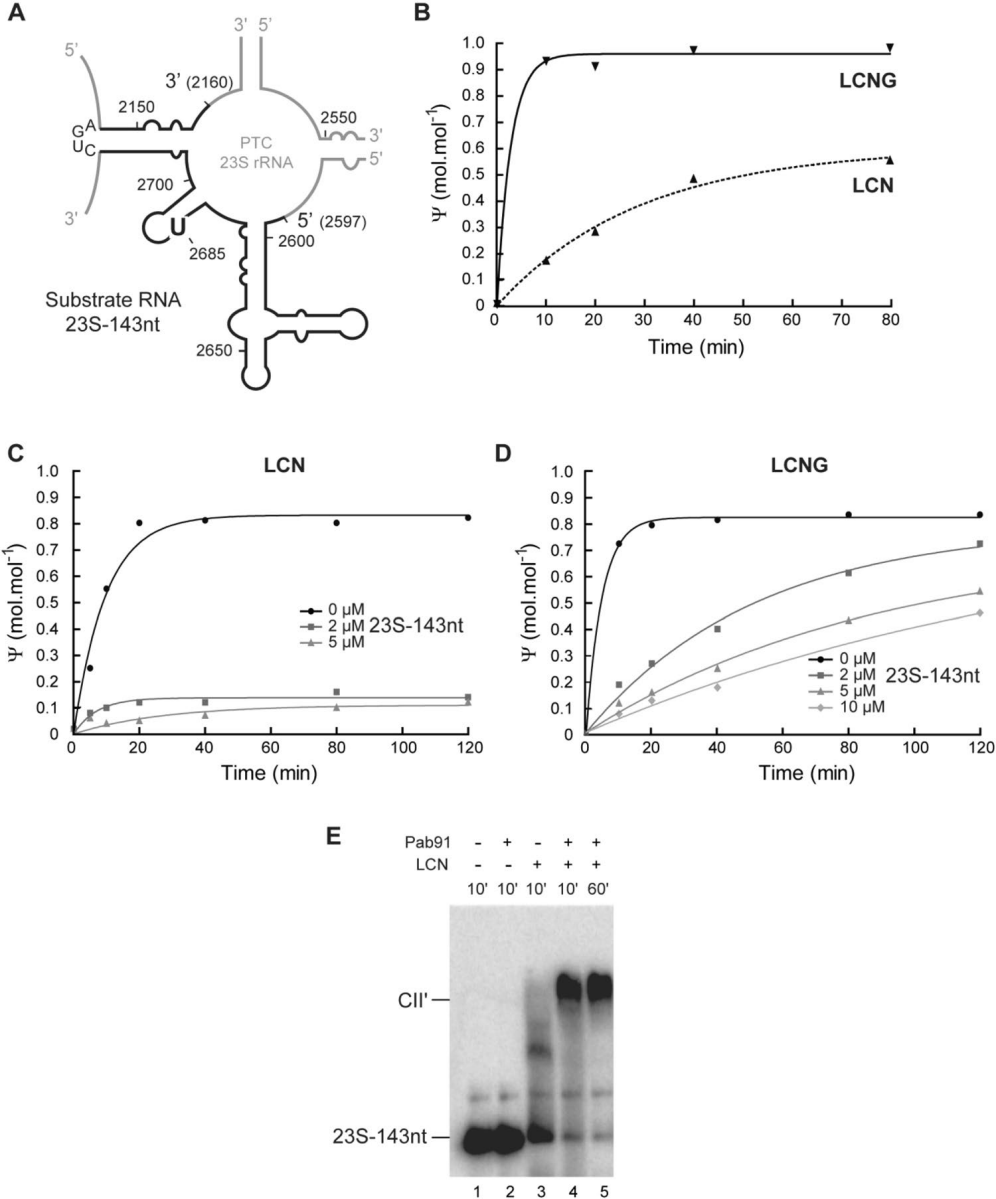
*In vitro* activity of *P*. *abyssi* Pab91 RNP enzyme for formation of Ψ_2685_ in a 143-nucleotide-long rRNA substrate. (**A**) The RNA substrate mimicking the peptidyl transferase center (PTC) region of the 23S rRNA. The secondary structure model of the *P*. *abyssi* PTC is represented. The U at position 2685 targeted by the Pab91 RNP is indicated. The region of RNA substrate 23S-143nt is delineated by the black line. The loop CUGA replaced a large portion of 23S rRNA corresponding to domains I, II, III, IV, VI and is used as a linker between the 5′ and 3′ parts of domain V. (**B**) Single-turnover activity of the *P*. *abyssi* Pab91 LCN and LCNG RNP enzymes for modification of radiolabeled 23S–143nt at 65 °C. The ^32^P was introduced in the phosphodiester bound preceding U_2685_ by a splinted ligation (detailed in Material and Methods). (**C**-**D**) Multiple-turnover activity of the LCN (**C**), and LCNG (**D**) enzymes for modification of radiolabeled 22–U substrate RNA at 65 °C. The unlabeled substrate RNA 23S-143nt was added in a 4-fold (2 μM), 10-fold (5 μM), or 20-fold (10 μM) excess over the RNP (~0.5 µM). A control was performed in absence of the unlabeled RNA (0 µM). (**E**) Electrophoretic mobility shift analysis (EMSA) of the binding of the radiolabeled substrate 23S–143nt with the Pab91 sRNP assembled with the L7Ae, Cbf5, and Nop10 mix. Incubation was performed at 65 °C during 10 and 60 minutes.

As the function of Gar1 in H/ACA sRNP activity had only been studied using short RNA fragments, we tested its activity on a long RNA substrate mimicking the 23S rRNA peptidyl transferase center (PTC). We engineered a DNA template allowing transcription of a 143-nucleotide long RNA fragment containing a large portion of the PTC domain (Fig. 4A). This substrate RNA designated 23S–143nt carries the U at position 2690(2685) in 23S rRNA that is targeted *in vivo* by the Pab91 sRNP, which was well characterized in our previous studies^31,35,55,65^. We verified that this substrate was modified *in vitro* at the target site by the *P*. *abyssi* Pab91 LCN enzyme under single-turnover conditions albeit less efficiently than the LCNG enzyme (Fig. 4B). However, the modification turnover was considerably diminished in the absence of Gar1 (Fig. 4C,D). The complex formed between the reconstituted Pab91 sRNP LCN and the radiolabeled 23S–143nt RNA substrate (complex CII’) was analyzed by electrophoretic mobility shift analysis^31^. Binding of this long substrate is efficient as high amounts of complex CII’ are formed (Fig. 4E, lane 4). The steady amounts of complex CII’ observed after 60 min of incubation (Fig. 4E, lane 5), are consistent with the absence of an efficient turnover of the RNP enzyme lacking Gar1.

### Modification at position U2589(2585) requires an RNA component and the H/ACA RNP proteins including Gar1

**Figure 5:**
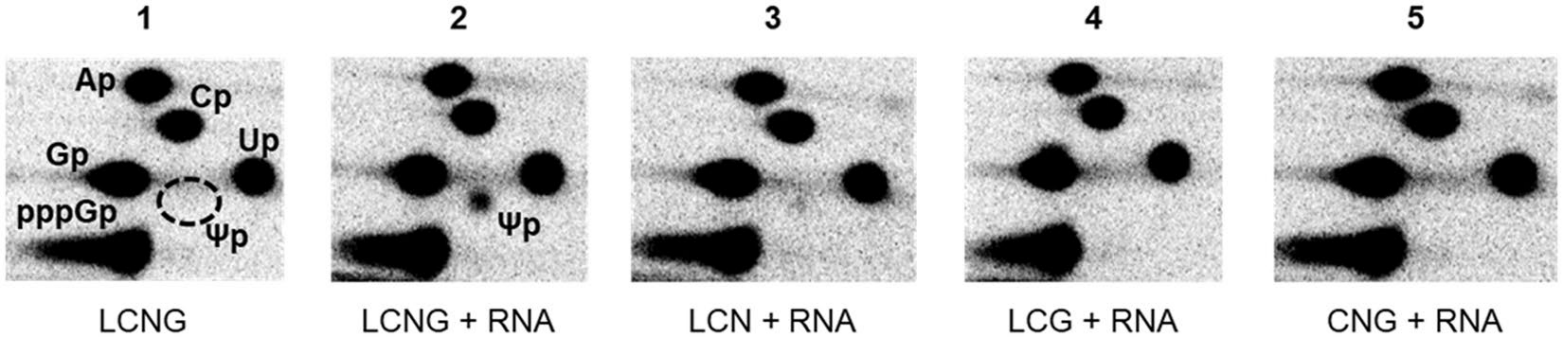
*In vitro* activity tests suggesting that Cbf5′s RNA dependent activity modifies orphan position U_2589(2585)_. The mini–23S–2589 RNA fragment was radiolabeled by incorporation of [α-^32^P]GTP during *in vitro* transcription. This substrate was incubated with different combination of the four recombinant proteins from *T*. *kodakarensis* L7Ae (L), Cbf5 (C), Nop10 (N), and Gar1 (G) and total RNAs extracted from a S100 fraction from KU216 cells (+RNA). After digestion with RNase T2, the amount of Ψ formation was estimated by two-dimension thin layer chromatography as described in Methods. The control reaction (panel 1) was performed in the presence of LCNG but without the addition of total RNAs. The plates corresponding to each panel were exposed together on a same screen of the PhosphorImager. The full-length image is presented in Supplementary Figure S5.

At this stage of the study, the modification Ψ_2589(2585)_ in 23S rRNA remained enigmatic. CMCT-RT analysis of rRNAs extracted from the Δ*cbf5* mutant indicated that Cbf5 is implicated in the formation of this modification (Fig. 3B, lanes 7 and 8), and that both Nop10 (Fig. 3B, lanes 11 and 12) and Gar1 (Fig. 3B, lanes 15 and 16) are required for this activity. The modification could either result from the non-RNA-guided activity of Cbf5 in complex with the two auxiliary proteins, or from the RNA-guided activity of Cbf5 present in a H/ACA sRNP enzyme with a still unknown RNA guide. In order to distinguish between these two possibilities, we tested (Fig. 5) the *in vitro* activity of the recombinant Cbf5 in the modification of the mini–23S–2589 RNA, whose sequence includes residues 2583 to 2599 of the 23S rRNA (Fig. 3A). Incubation of the radiolabeled mini–23S–2589 with the combination of the four recombinant proteins Cbf5, Nop10, Gar1, and L7Ae did not allow formation of Ψ. However, we observed partial conversion of U_2589_ into Ψ (Ψ ~0.14 mol^−1^.mol^−1^) when total RNAs extracted from *T*. *kodakarensis* cells were added to the protein mix. This activity requires the presence of the full set of proteins Cbf5, Nop10, Gar1, and L7Ae. We hypothesize that modification at position U_2589_ in 23S rRNA is generated by an RNP that is assembled by association of proteins L7Ae, Cbf5, Nop10, and Gar1 with a still unknown guide sRNA present in the *T*. *kodakarensis* total RNAs.

## Discussion

Genetic manipulation by gene disruption and functional complementation is an extremely valuable tool for the study of metabolic processes in a living cell. Such methods have been widely applied in bacterial or eukaryotic model organisms and have enable the characterization of numerous modification enzymes acting on RNAs in *E*. *coli* and *S*. *cerevisiae*^66,67^. However, archaeal organisms were not included in this thorough-full characterization since targeted gene deletion in most of those organisms is still a rather difficult task. Genetic manipulations have only been used in few archaeal species such as Thermococcales, Sulfolobales, Methanogens, and Halophiles^68–72^. In the RNA modification research field, gene deletion has only been performed in halophilic *Haloferax volcanii* for tRNA modification enzymes^73^, for 2′-*O*-methylation guide box C/D RNA^74^ and for several PUS including Cbf5 and Pus10^50^. Establishment of the *pyrF*^*-*^ KU216 *T*. *kodakarensis* strain and the use of *pyrF* as a marker to select uracil auxotrophic cells enabled the development of a strategy for targeted gene disruption in this sulfur reducing, strictly anaerobic hyperthermophilic organism^57–59,75^. By applying this system to *T*. *kodakarensis*, we generated the first disruption mutants of the two PUS enzymes Cbf5 and Pus10, and of the Gar1 and Nop10 components of the catalytic H/ACA RNPs of a hyperthermophilic archaea. These mutants make it possible to analyze the activity of the modification machinery at high temperatures and to study the effect of RNA modifications in these peculiar growth conditions. Up to now, the *in vitro* specificity and turnover of the H/ACA sRNP enzymes from *P*. *abyssi* have been shown to rely on high temperatures^39^ and *in vivo* data have not been documented.

In contrast to eukaryotes^76^, but in agreement with observations made in halophilic archaeon *H*. *volcanii*^50^, the *T*. *kodakarensis* gene encoding Cbf5 homolog is dispensable. However, deletion of *cbf5* led to a marked decrease in growth rate of *T*. *kodakarensis* cells at 90 °C (Fig. 2). It was previously observed that deletions of genes encoding modification enzymes belonging to the same PUS family as Cbf5, e.g. *truB* in *E*. *coli*^67,77^ or in the thermophilic eubacterium *Thermus thermophilus*^78^, are not lethal either, but are associated with thermosensitive phenotypes. The thermosensitive slow growth phenotype of Δ*cbf5* mutant can be rationalized by considering that lack of modification of one subset of RNAs modified by Cbf5, e.g. tRNAs and/or rRNAs, results in reduced RNA stability or reduced ribosome activity at temperatures higher than the optimal growth temperature. It is also conceivable that Cbf5 has a function that is not related to pseudouridylation but that contributes to growth in these conditions. Surprisingly, deletions of the H/ACA RNP components Gar1 and Nop10 led to a slow growth phenotype at all the temperatures tested, i.e. 75 °C, 85 °C and 90 °C (Fig. 2). These data suggest that these proteins may have additional functions not associated with Cbf5 and H/ACA RNPs.

The null mutant of the PUS Pus10 showed marked phenotypes with a decrease of the growth rate at all the temperatures tested and formation of aggregates in liquid culture (Supplementary data, Fig. S3). These data suggest Pus10 contributes more than Cbf5 to cell growth at high temperatures. However, in contrast to *H*. *volcanii*^50^, Pus10 from *T*. *kodakarensis* is not essential for cell viability. The essential role of Pus10 in *H*. *volcanii* may be either due to its tRNA modification activity, i.e. the formation of tRNA Ψ_54_ and Ψ_55_, one or both of these modifications may be crucial for the cell viability of this halophilic archaeon. Alternatively, in this organism, Pus10 could also modify other cellular RNAs or have a function that is not related to pseudouridylation.

In the future, it would be of interest to extend the analysis of the null mutants produced to compare the Cbf5 and Pus10 activities at positions 54 and 55 in elongator tRNAs. This would make it possible to determine whether Cbf5 is simply needed to compensate for occasional Pus10 activity defects, as the tRNA Ψ_55_ nucleotide is extremely important to stabilize the tertiary structure of tRNA^79^. Isolation of the double mutant Δ*cbf5*Δ*pus10* would also be helpful for these studies.

The present work confirms the rather large substrate specificity of Cbf5 in archaea. This multifunctional PUS modifies cellular RNAs using two independent, but complementary mechanisms: (i) an activity linked to its inclusion in H/ACA sRNPs complexes together with L7Ae, Nop10, and Gar1 and one H/ACA sRNA^30,31^, (ii) as a protein stand-alone enzyme acting alone or in complex with auxiliary proteins Nop10 and/or Gar1^55^. We previously demonstrated that in addition to tRNA U_55_, U_2603_ in *P*. *abyssi* 23S rRNA represents a possible second substrate for the non RNA-guided activity of Cbf5 in *P*. *abyssi*^55^. Here, we found that the modification is conserved in *T*. *kodakarensis* 23S rRNA, i.e. at position U_2607_ (Fig. 3B). Modification at this position likely implies that at some stage of its maturation, the rRNA sequence mimics the folding of the tRNA TΨC arm. Our comparison of the Ψ status in wild-type, Δ*cbf5* and Δ*pus10* null mutants clearly demonstrates (Fig. 3B) that in *T*. *kodakarensis*, there is no redundancy between the Cbf5 and Pus10 activities for modification of U_2607_ in 23S rRNA. It only relies on Cbf5.

The present data also enlarge the catalog of sites modified by Cbf5 to position U_2589_ in 23S rRNA. Our observation that total RNA fraction from *T*. *kodakarensis* restores Ψ_2589_ formation *in vitro* supports a possible role for an RNA component likely acting as a guide RNA for the activity of a catalytic RNP enzyme. Analysis of the 23S rRNA extracted from each disrupted mutant strain demonstrated the implication of each of the three *cbf5*, *nop10*, and *gar1* genes encoding the H/ACA sRNP components for Ψ_2592_ (used as control) as well as for Ψ_2589_ formation (Fig. 3B). We did not succeed in isolating a *l7ae* null mutant. This is not surprising, as in addition to being a component of H/ACA RNPs, L7Ae is a ribosomal protein. Nevertheless, we showed that L7Ae is required for Ψ_2589_ formation *in vitro* (Fig. 5). This adds credit to the assumption that a fully active RNP enzyme could be formed when L7Ae and the three other protein components Cbf5, Nop10, and Gar1 of box H/ACA sRNP assembled with a guide RNA present in the total RNA fraction extracted from *T*. *kodakarensis* cells. In a previous work, we identified six H/ACA sRNAs by bioinformatics analysis of the *P*. *abyssi* genome and found that none of the *in vitro* sRNPs assembled with each of these RNA guides catalyzes Ψ_2585_ formation, i.e. modification of the U equivalent to position 2589 in *T*. *kodakarensis* 23S^55^. To identify the guide RNA potentially involved in Ψ_2589_ formation, we fractionated *T*. *kodakarensis* total RNAs on acrylamide gel, eluted RNA fractions from gel slices and tested if they could complement L7Ae, Cbf5, Nop10, and Gar1 for Ψ_2589_ formation *in vitro*. An active fraction was indeed identified and its RNA content was analyzed by RNASeq. However, no new RNAs with the known features of archaeal box H/ACA sRNAs were detected suggesting that the RNA required for the Ψ_2589(2585)_ formation could correspond to a new type of guide RNA. Several RNA candidates need to be individually tested in *in vitro* modification assays before generating a null mutant of the gene sequence of the identified RNA for *in vivo* analysis.

We show that both *in vivo* and *in vitro*, unlike Nop10, Gar1 is absolutely indispensable for the RNA-independent activity of Cbf5 at position 2607(2603) in archaeal 23S (Fig. 3B,C). The work by R. Gupta’s team already outlined differential roles of *Methanocaldococcus jannaschii* Nop10 and Gar1 for Ψ_55_ formation in tRNA by Cbf5 *in vitro*^44^. Differential roles of Nop10 and Gar1 were also observed in H/ACA RNP activity. Nop10 is a key component of RNP architecture and activity^30,31,35^. Gar1 helps position the thumb to open and close the active site of Cbf5^38^. However, Gar1 is dispensable for total modification of an excess of substrate RNA in single-turnover reaction catalyzed by H/ACA RNPs^31^. Nevertheless, under these conditions the presence of Gar1 enhances the kinetics of the reaction^30,31,38,39^. Interestingly, the critical contribution of Gar1 to the turnover of an H/ACA RNP is also evidenced by the activity test performed *in vitro* in this study on the large 23S rRNA fragment of domain V (Fig. 4). Gar1 may thus play equivalent role in Cbf5 RNA-dependent and RNA-independent activities. In particular, it may stimulate the turnover of Cbf5 for rRNA Ψ_2607(2603)_ formation. As an extrapolation, we can also suggest that the RNA-independent activity relies on the presence of Gar1 to facilitate the binding of Cbf5 to the RNA and/or to allow catalytic activity of the enzyme. Structural information on the [Cbf5:substrate RNA] complex formed in the presence and absence of Gar1 and comparison with the structure of the [H/ACA RNP:substrate RNA] complex^38^ might provide further information on the exact roles played by Gar1 in the RNA-dependent and RNA-independent activities of Cbf5.

## Conclusion

We present here the analysis of several proteins involved in pseudouridylation of ribosomal rRNA in *T*. *kodakarensis*. We demonstrate that Cbf5 is responsible for Ψ formation at multiple positions in the large subunit rRNA. Cbf5 performs this function as the catalytic subunit of RNPs and modifies at least one position with an RNA guide-independent activity. Pus10 does not appear to be involved in rRNA modification. Gar1 contributes to each of the activities of Cbf5 characterized *in vivo*.

## Methods

### Strain and culture

Cells of the *T*. *kodakarensis* strain KU216, lacking *pyrF* gene (KU216 *pyrF*^*-*^) were cultivated under anaerobic conditions at the optimal growth temperature of 85 °C in a nutrient-rich medium (ASW-YT) containing 0.5% yeast extract, 0.5% tryptone and artificial sea water as described previously^58,59^. Other growth temperatures (75 °C and 90 °C) were also used to study the phenotypic effects of the deletions. ASW-CH medium was used for the selection based on uracil autotrophy as described previously^57^.

### Sequence analysis and homology search

*T*. *kodakarensis* putative PUS genes were identified in a homology search with NCBI BLAST^80^, using the amino acid sequence of *Pyrococcus abyssi* PUS Pab0356 (*cbf5*) and Pab2391 (*pus10*), and H/ACA sRNP components Pab7213 (*nop10*), Pab3084 (*gar1*), Pab0460 (*l7ae*) as query. Sequence analysis of *T*. *kodakarensis* was performed using the NCBI genomic database (http://www.ncbi.nlm.nih.gov) and the BAGET sequence exploration tool (http://archaea.u-psud.fr/bin/baget.dll/EXEC). To ascertain the identity of the identified ORFs, sequences of *T*. *kodakarensis* proteins were compared with already known and biochemically characterized PUS and H/ACA sRNP proteins from *P*. *abyssi*, *P*. *furiosus*, or *Haloferax volcanii*. Only Pus10/Cbf5 showed slight similarity, and Nop10 and L7Ae shared limited similarity with IF2-gamma and L30e, respectively (see Supplementary data, Table S1). Inspection of the entire genome of *T*. *kodakarensis* revealed no orthologs of any other potential proteins (Supplementary data, Table S2).

### Construction of the disruption vectors

The plasmids used for gene disruption in *T*. *kodakarensis* were constructed as follows. DNA fragments containing the sequence of the ORF to be deleted along with about 1 kb of each 5′ and 3′ flanking regions were amplified by PCR, using specific primer pairs (Primers F and R in Supplementary data, Table S3) and genomic DNA from the KU216 strain as template. The PCR-amplified fragments were cloned into the pGEM-T easy vector (Promega). Each recombinant clone was confirmed by full sequencing of the fragments. Inverse PCR reactions were performed with InvF and InvR primer pairs (Supplementary data, Table S3) to amplify the flanking regions of each target gene along with the vector backbone. The PCR fragments were ligated by T4 DNA ligase, giving a continuous DNA fragment containing both flanking sequences without the target gene. The resultant DNA fragments were excised with the appropriate restriction enzymes and inserted into the pUD2 vector. This vector is a pUC118-based plasmid harboring the *pyrF* gene as a nutrient marker^58^.

### Construction of the gene deletion mutants of *T. kodakarensis*

The knockout strains were obtained with the gene targeting method for *T*. *kodakarensis*^57,58^. KU216 *pyrF*^-^ cells were transformed with each of the disruption vectors. After transformation, the *pyrF*^+^ transformants showing uracil prototrophy were selected by cultivating the cells in the ASW-CH medium lacking uracil. Then the *pyrF*^+^ cells were plated on ASW-YT containing 0.85% (w/v) of 5-fluoroorotic acid (5-FOA) for selection of uracil auxotrophic cells produced by internal chromosomal recombination. The colonies were picked and cultivated in 5 mL ASW-YT. Genomic DNA of each culture was isolated by phenol/chloroform extraction, followed by isopropanol precipitation. The genotypes of isolated strains were validated by whole genome sequencing of each mutant.

### Whole genome sequencing of *T*. *kodakarensis* DNA

Genomic DNA from *T*. *kodakarensis* mutant strains was extracted following the standard procedure^81^. About 100 ng of DNA in 130 µl of water was fragmented using Covaris M200 AFA ultrasonicator to fragments of ~200 bp. Sonication efficiency was confirmed by capillary electrophoresis using HS DNA chip (Agilent). DNA fragments were converted to sequencing libraries using NEBNext® Ultra™ II DNA Library Prep Kit, according to the manufacturer’s recommendations. Amplified libraries were verified by capillary electrophoresis and quantified by fluorometry using QuBit. Sequencing was performed in SR50 and/or PE2x100 mode, using Illumina HiSeq1000. After trimming of the adapter sequences, clean reads were aligned to the reference genome (GCF_Tkodakarensis_ASM996/ATGC280) using Bowtie2 in*–local-sensitive* mode. Counting of the reads’ coverage was done using bedtools and the associated gff3 file.

### Total RNA isolation

Growth of *T*. *kodakarensis* cells was stopped at the end of the exponential phase. Total RNA was isolated as described previously^82^. The cells were centrifuged and washed in 0.8x artificial sea water. The pellets (~10^9^ cells) were dissolved in extraction buffer (4 M guanidine thiocyanate, 25 mM sodium citrate pH 7; 0.5% *N*-lauroylsarcosine and 0.1 M β-mercaptoethanol) and RNAs extracted by phenol/chloroform/isoamyl alcohol followed by ethanol precipitation, dissolved in H_2_O and stored at −80 °C. For RNAs extracted from the S100 fraction, the cells were resuspended in sonication buffer (25 mM Tris-HCl pH 7.5, 5 mM MgCl_2_, 25 mM KCl, 2 mM DTT, and 10% glycerol), treated by ultra sounds, the concentration of KCl was adjusted to 150 mM before centrifugation at 16,000 × g for 30 min. The S100 extract was obtained after centrifugation at 100,000 × g for 1 hour and stored in 20% glycerol at −80 °C. RNAs from the S100 fraction were extracted with phenol/chloroform/isoamyl alcohol followed by ethanol precipitation, dissolved in H_2_O and stored at −80 °C.

### Mapping of pseudouridine (Ψ) residues in 23S rRNA

The N-cyclohexyl-N′-(2-morpholinoethyl)-carbodiimide metho-p-toluenesulfonate (CMCT) modification protocol^82^ was adapted from^83^. Briefly, 10 µg of total RNAs were modified by CMCT, which preferentially reacts with U and Ψ residues. Extended incubation of the CMC-modified RNAs at alkaline pH (10.4) allows the hydrolysis of U–CMC adducts, which are less stable than the Ψ–CMC adducts. The positions of Ψ–CMC adducts were then detected by primer extension using avian myeloblastosis virus (AMV) reverse transcriptase (MP Biomedicals) and 5′–end labeled primer O-2941 (5′-CGTTCCCCTTTAATGGGTGA-3′) complementary to 23S rRNA sequence. The synthesized cDNAs were fractionated by electrophoresis on a 7% sequencing gel which was analyzed by autoradiography.

### Recombinant protein production

The recombinant proteins Cbf5, Gar1, Nop10, and L7Ae were produced in strain *E*. *coli* C41 (DE3) pRare2 as GST-fusion proteins from recombinant pGEX-6P1 plasmids, and purified as described previously^31,39^. To ensure removal of contaminating nucleic acids from *E*. *coli*, the purification protocol includes incubation with polyethylenimine (PEI)^39^. Circular dichroism (CD) spectroscopy was used to control the absence positive Δε values in the spectral region above 240 nm, which is specific of CD spectra measured for RNAs^35^ (Supplementary data, Fig. S6).

### Production of RNA transcripts

DNA template used for *in vitro* transcription of the *P*. *abyssi* Pab91 guide RNA was obtained by PCR amplification with the forward primer generating the sequence of the T7 promoter^31^. The DNA fragments carrying the T7 RNA polymerase promoter sequence upstream of the sequence encoding the 23S rRNA sequence targeted by Pab91 and corresponding to the 22–U substrate RNA^39^, or encoding the two 23S rRNA fragments mini–23S–2589 and mini–23S–2607 were obtained by annealing of complementary DNA oligonucleotides. The DNA template encoding the substrate rRNA 23S–143nt was generated by multiple PCR amplifications. In this construct nucleotide at position 2717 in *P*. *abyssi* 23S rRNA was connected to nucleotide 2143 by the 5′-CTGA-3′ sequence. The obtained fragments were cloned in vector pCR2.2 and their sequences verified by sequencing.

Small fragments were transcribed using the MEGAshortscript T7 transcription kit (Invitrogen). For the other fragments, the transcription reactions were carried out as previously described^31,65^. The RNA transcripts were purified on 8 M urea-PAGE, and stored in H_2_O at −80 °C.

The substrate RNA 22–U used for Pab91 sRNP activity measurements was radiolabeled during transcription in presence of [α-^32^P]CTP. The substrates mini–23S–rRNA–2589 and mini–23S–rRNA–2607 used to measure Cbf5 activity were radiolabeled during transcription in presence of [α-^32^P]CTP and [α-^32^P]GTP, respectively. Radiolabeling of the phosphodiester linkage 3′ to the targeted U_2685_ in the substrate RNA 23S–143nt was obtained by splinted ligation of two *in vitro* transcribed RNA fragments of the 23S rRNA^84^. Briefly, 10,000 cps of the 5′ end radiolabeled donor RNA spanning 23S rRNA positions 2686 to 2160 and containing the CUGA sequence connecting position 2717 with position 2143 were mixed with 100 pmol of the acceptor RNA spanning positions 2603 to 2685, in the presence of 50 pmol of a DNA single-stranded splint, which was perfectly complementary to the sequence of the donor and acceptor RNAs, in 50 mM Tris-HCl pH 7.4 containing 10 mM of MgCl_2_, 2 nmol of ATP, and 2 nmol of DTT. Hybridization of the two RNAs with the DNA splint was obtained by incubation at 75 °C for 4 min and a slow cooling to room temperature. The ligation reaction was achieved with 75 U of T4 DNA ligase (Fermentas) at 4 °C overnight. The DNA splint was digested by adding 3 U of DNase RQ1 (Promega) at 37 °C for 30 min. The radiolabeled ligation products were purified on a 8 M urea-PAGE (6%).

### Assembly of the LCN and LCNG RNP enzymes

LCN Pab91 RNP enzyme assembly was obtained by mixing the sRNA Pab91 and the proteins L7Ae, Cbf5, and Nop10 as previously described^65^. Protein Gar1 was added to this mix to assemble the LCNG RNP enzyme.

### Analysis of substrate RNA binding to the Pab91 sRNP

Complex CII’ was obtained by association of the sRNA present in the sRNP containing proteins L7Ae, Cbf5, and Nop10 (LCN) with radiolabeled substrate RNA, as described previously^31,35,39^. The radioactivity present in the CII’ complex or in the unbound fraction was revealed using a Phosphorimager (Typhoon 9410, Amersham Biosciences).

### RNA:Ψ-synthase activity measurements in single- and multiple-turnover conditions

To test for the RNA:Ψ-synthase activity, we used an approach based on the nearest-neighbor analysis^65^. Conditions for time course analysis of single-turnover reactions by reconstituted Pab91 sRNP particles LCN and LCNG were previously detailed^31,35,39,48^. Briefly, ~4 pmol of unlabeled Pab91 sRNA and ~150 fmol of labeled 22–U substrate RNA were mixed with protein combination L7Ae–Cbf5–Nop10 or L7Ae–Cbf5–Nop10–Gar1 (200 nM each) at 65 °C. In these conditions, the concentration for the reconstituted sRNP was estimated to be of ~0.5 µM. For multiple turnover reactions, an excess of unlabeled substrate RNA 23S–143nt, ranging from 1 to 10 μM, was added to the reaction mix.

The guide RNA was omitted to perform single turnover reactions for the measurement of the RNA guide-independent activity of Cbf5. The radiolabeled substrates mini–23S–rRNA–2589 and mini–23S–rRNA–2607 (~150 fmol) were incubated with different combination of the recombinant proteins Cbf5, Nop10, Gar1, and L7Ae (200 nM each).

For all these assays, aliquots were collected at several time points and the reaction was stopped by phenol/chloroform extraction followed by ethanol precipitation. The recovered RNAs were digested with ~0.4 U of RNase T2 to generate 3′-monophosphate nucleotides that were fractionated by thin layer chromatography (TLC)^85^. For the reactions performed on the RNA 22–U, one dimension chromatography was sufficient to fractionate the labeled nucleotides whereas two-dimensional TLC is needed for activity measurement with mini–23S–rRNA–2589 or mini–23S–rRNA–2607. The radioactivity of the 3′-monophosphate nucleotides was quantified with the Phosphorimager and quantified using the ImageQuant software Version 5.2 (Molecular Dynamics). The quantities of Ψs formed were determined by taking into account the total number of U nucleotides in the substrate RNA. The obtained values in moles of Ψ per mole (mol.mol^−1^) of substrate RNA were analyzed with Prism software Version 5.04 (GraphPad).

## Electronic supplementary material

Dataset 1

## Data Availability

All data generated or analyzed during this study are included in this published article (and its Supplementary Information files). Raw sequencing data are available in European Nucleotide Archive under accession number PRJEB28421.
