# Supplementary material for: Contribution of protein Gar1 to the RNA-guided and RNA-independent rRNA:Ψ-synthase activities of the archaeal Cbf5 protein
Source: Sci Rep. 2018 Sep 14;8:13815. doi: 10.1038/s41598-018-32164-0 (PMC6138745; doi:10.1038/s41598-018-32164-0)
Supplement: Supplementary file 1 — Dataset 1 [file 41598_2018_32164_MOESM1_ESM.pdf]

Supplementary data

Contribution of protein Gar1 to the RNA guided and RNA-independent rRNA:Ψ-synthase activities of the archaeal Cbf5 protein

Ryosuke Fujikane [1,2,4], Isabelle Behm-Ansmant [1], Anne-Sophie Tillault [1,5], Christine Loegler [1], Valérie Igel-Bourguignon [1], Evelyne Marguet [2], Patrick Forterre [2], Christiane Branlant [1], Yuri Motorin [1,3], and Bruno Charpentier [1*]

[1] Université de Lorraine, CNRS, Ingénierie Moléculaire et Physiopathologie Articulaire (IMoPA), F-54500 Nancy, France.
[2] Institut de Génétique et Microbiologie (IGM) Bat 409, Université Paris-Sud, Centre d'Orsay, 91405 Orsay Cedex, France.
[3] Université de Lorraine, CNRS, INSERM, IBSLor, F-54500 Nancy, France.

[4] Present address: Fukuoka Dental College, Dept. of Physiological Sciences and Molecular Biology, Section of Cellular and Molecular Regulation, 2-15-1 Tamura, Sawara-ku, Fukuoka, 814-0193 Japan.
[5] Present address: Department of Biological Sciences, University of Lethbridge, Lethbridge, Alberta, Canada.

* Correspondence: bruno.charpentier@univ-lorraine.fr; Tel.: +33 3 72 74 66 27

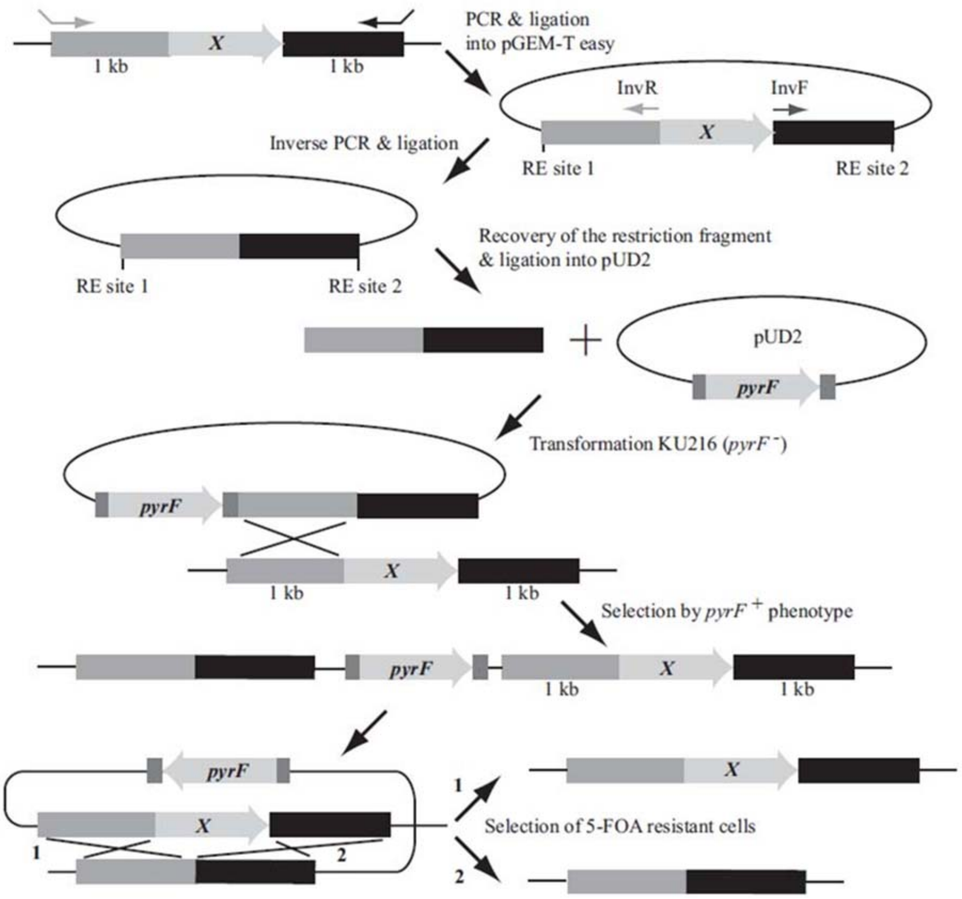

Figure S1. Scheme of the strategy used for ORF deletion in *T. kodakarensis* (adapted with permission from Fujikane *et al. Genes Genet. Syst*. **85**, 243-257). The target gene X is in gray. Arrows with flap represent primers used for PCR amplification of the target gene and 1 kb of each flanking regions. The obtained PCR fragment was cloned, and the resulting recombinant vector was used as the template for a PCR reaction performed with primers InvR and InvF represented by horizontal arrows. After ligation, the resulting PCR fragment corresponded to a continuous DNA fragment containing both flanking sequences without the target gene. The fragment was sub-cloned in vector pUD2 harboring *pyrF* as a selectable marker. The recombinant pUD2 vector was used to transform *T. kodakarensis* KU216 strain lacking the *pyrF* gene. The *pyrF*$^{+}$ transformants showing uracil prototrophy were selected. Intra-chromosomal recombination between homologous flanking regions were selected by plating cells on 5-fluoroorotic acid (5-FOA)-containing medium. Reversion to WT corresponded to recombination between regions 1, whereas recombination between regions 2 led to gene X disruption.

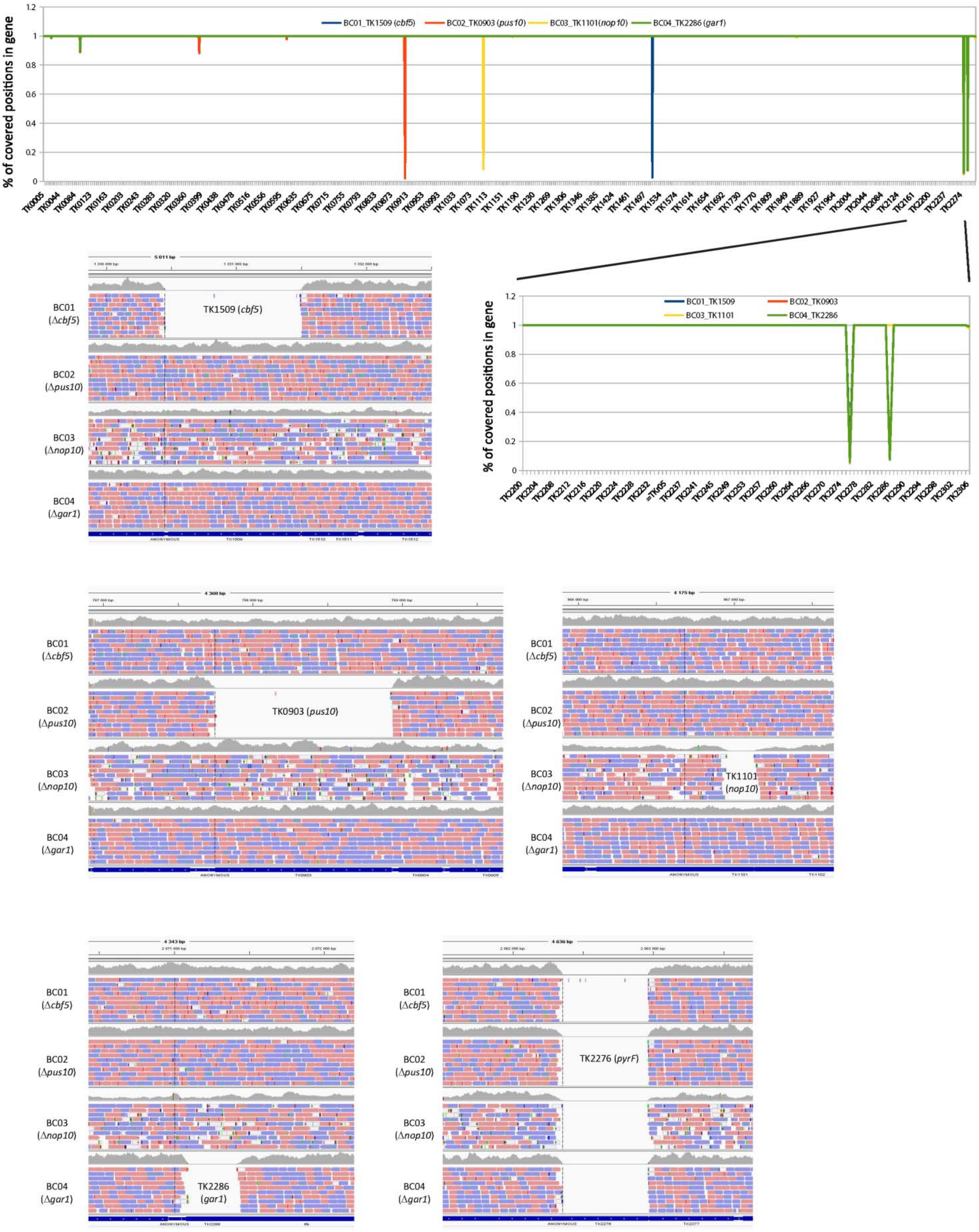

Figure S2. Percentage of covered positions for whole genome of *T. kodakarensis*. Coverage was calculated position by position and intersected with coordinates of every ORF of the genome. Inset shows the region encompassing TK2200–TK2306, which contains deletions of *pyrF* (TK2276) and *gar1* (TK2286). Individual panel shows screenshots of IGV genomic viewer for each concerned region in the four deleted strains.

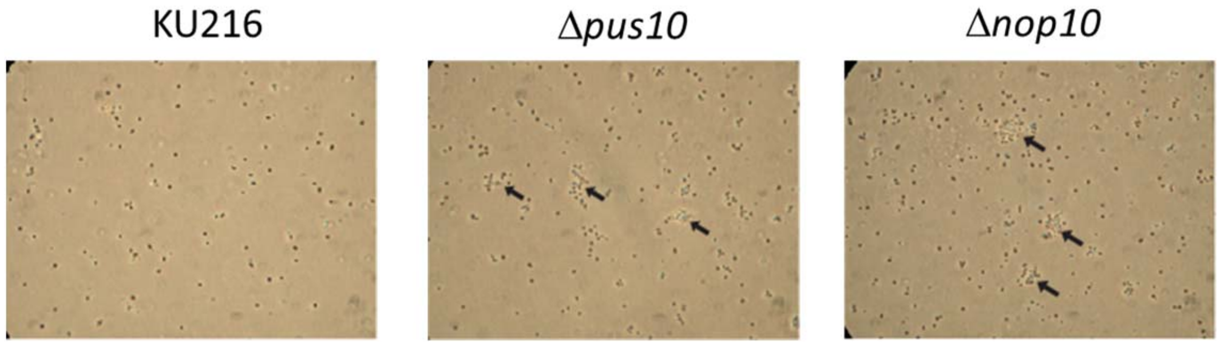

Figure S3. Optical microscope observations showing cell aggregation (indicated by arrows) in mutant strains Δ*pus10* and Δ*nop10.* KU216: wild type cells.

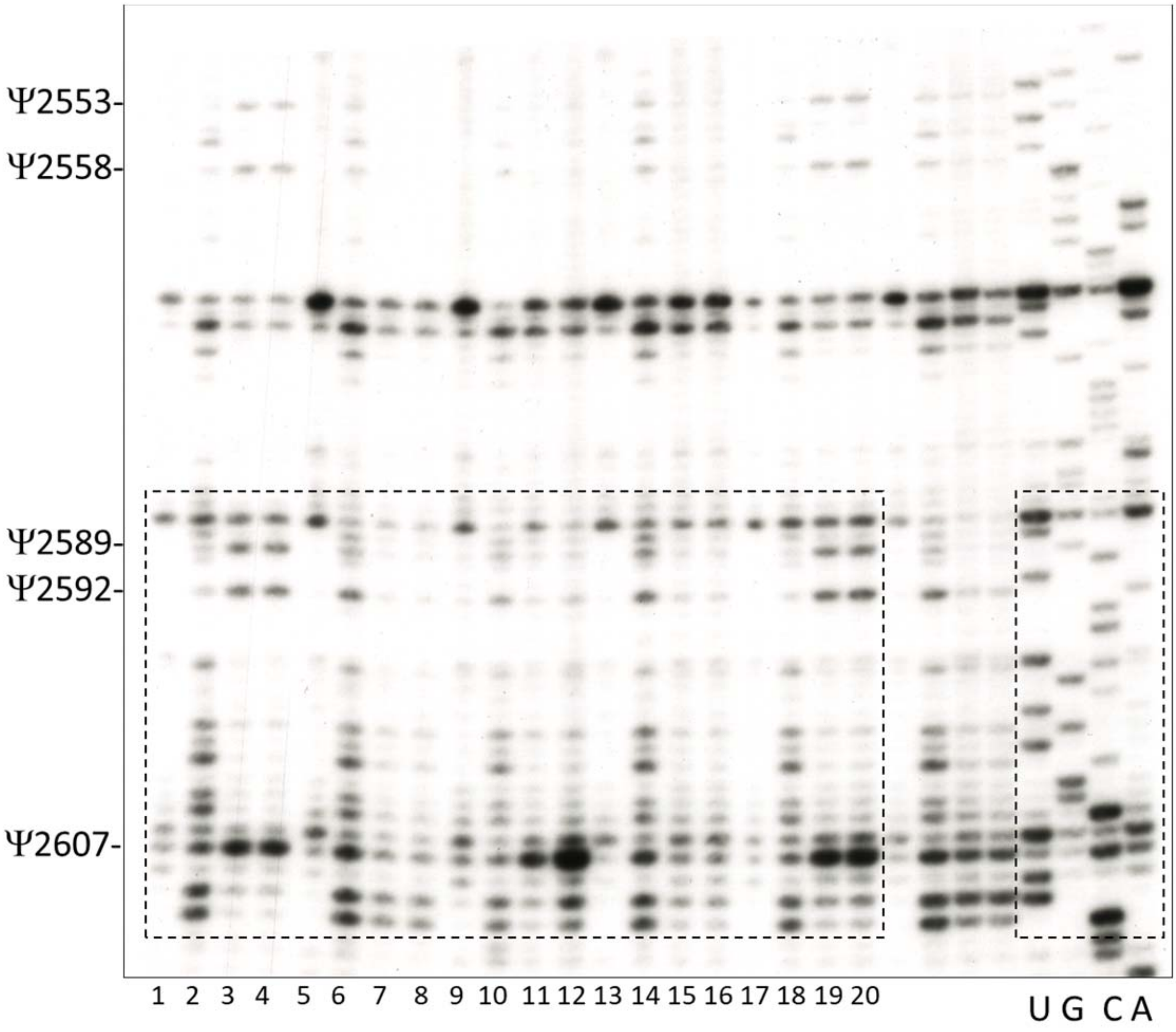

Figure S4. Full-length gel containing the panels shown in Fig. 3B. The different locations of the gel that were used to construct panel B are boxed with a dashed line. Samples in lanes between lane 20 and lane U not related to this work are not shown in the final figure.

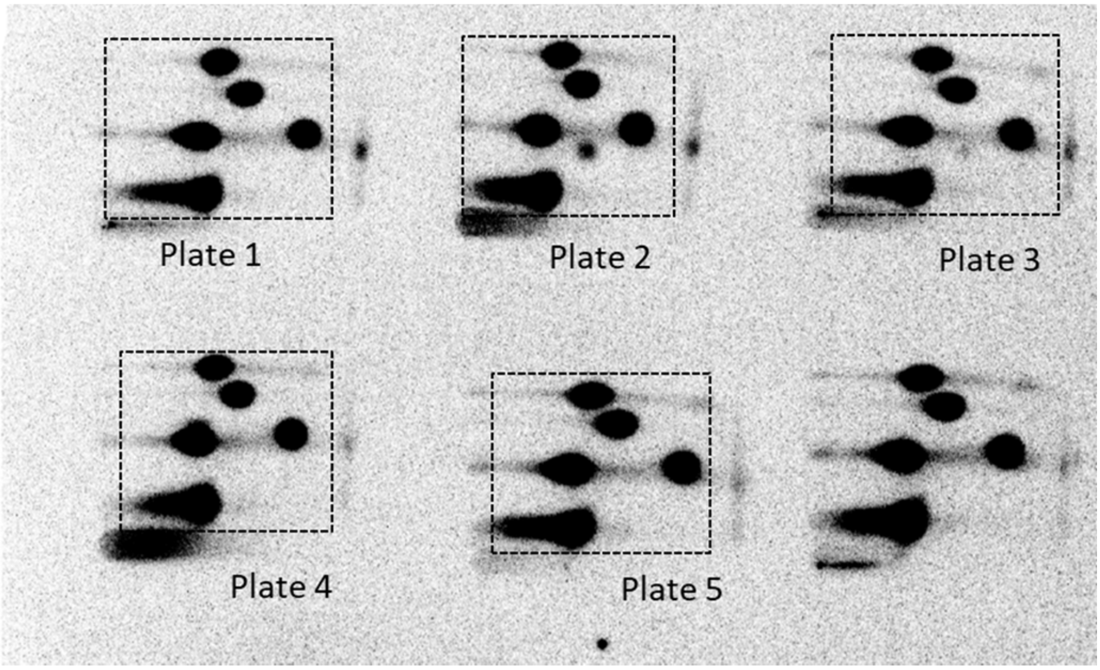

Figure S5. Full-length image obtained after Phosphorimager analysis of the 5 TLC plates displayed in Fig. 5. These plates were exposed together on a same screen. The locations on each plates used to construct Fig. 5 are demarcated by the dashed lines.

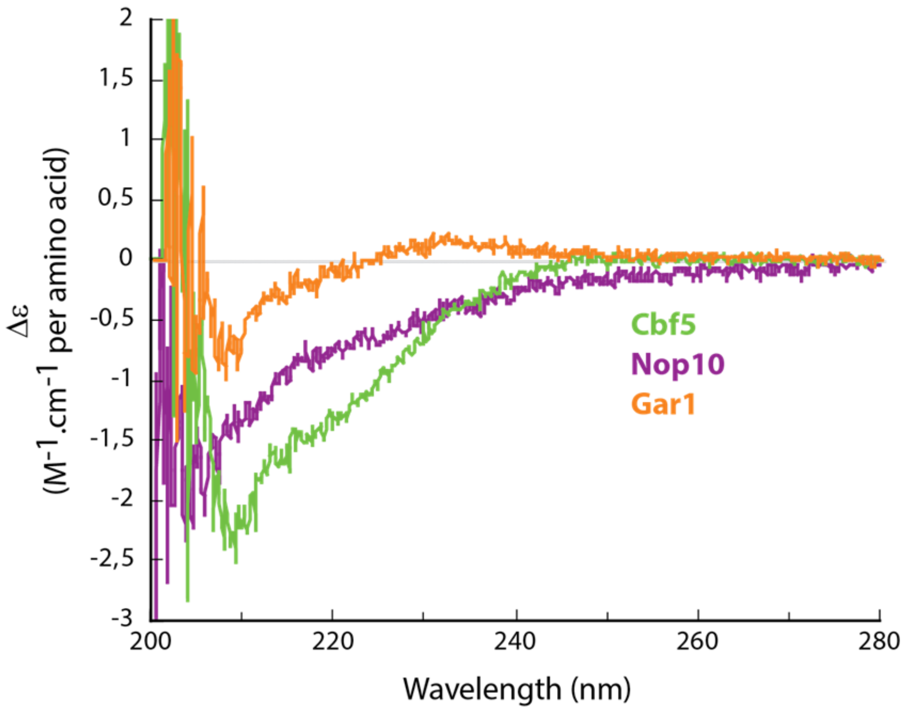

Figure S6. Circular dichroism spectroscopy of purified recombinant proteins. CD spectra from 200 to 280 nm were recorded as previously detailed (Fourmann *et al*., 2013) for Cbf5 (in green), Nop10 (in purple), and Gar1 (in orange). Proteins were individually diluted to a concentration of approximatively 2 µM in a buffer containing 10 nM Tris HCl (pH 7.5), 200 nM NaCl, 5 mM $MgCl_2$, and 1 mM DTT, and placed into a quartz cuvette with 0.875 cm path length at 25°C. Three repeats spectra were averaged for each protein.

Table S1. Sequence analysis of PUS Cbf5 and Pus10 and H/ACA sRNP protein components Nop10, Gar1, and L7Ae. Sequences of *T. kodakarensis* proteins were compared with already known and biochemically characterized proteins from *P. abyssi, P. furiosus,* or *Haloferax volcanii*.

Characterized PUS	Organism	Reference	*T. kodakarensis* ORF	Identity %	p-value, RefSeq *T. kodakarensis* database
Pus10 (WP_011012279.1)	*P. furiosus*	PMID:16920741	Pus10 (TK0903) BAD85092	64%	$7x10^{-180}$
idem			Cbf5 (TK1509) BAD85698	29%	0.054
Pus10 (PsuX) HVO_1979	*H. volcanii*	PMID: 21628430	Pus10 (TK0903) BAD85092	39%	$1x10^{-78}$
idem			Cbf5 (TK1509) BAD85698	29%	0.035
Cbf5 (WP_010867646.1)	*P. abyssi*	PMID:15933208	Cbf5 (TK1509) BAD85698	91%	0.0
idem			Nol1/Nop2(WP_011251072)	39%	$7x10^{-9}$
Cbf5 (HVO_2493)	*H. volcanii*	PMID: 21628430	Cbf5 (TK1509) BAD85698	46%	$3x10^{-84}$
idem			Pus10 (TK0903) BAD85092	27%	0.80
Nop10 (WP_010867970.1)	*P. abyssi*	PMID:15933208	Nop10 (TK1101) BAD85290.1	88%	$3x10^{-35}$
			IF2-gamma (WP_011250896.1)	54%	$2x10^{-5}$
Gar1 (WP_010867430.1)	*P. abyssi*	PMID:15933208	Gar1 (TK2286) BAD86475	67%	$7x10^{-40}$
L7ae (CAB49588.1)	*P. abyssi*	PMID:15933208	L7ae (TK1311) BAD85500	93%	$3x10^{-79}$
			L30e (WP_011250031.1)	31%	$3x10^{-5}$

Table S2. Search for potential orthologs of PUS Cbf5 and Pus10 and H/ACA sRNP protein components Nop10, Gar1, and L7Ae in *T. kodakarensis*.

T. kodakarensis gene	Ortholog in *T. kodakarensis* genome	Identity, %	p-value, RefSeq *T. kodakarensis* database
Pus10 (TK0903) BAD85092	WP_011249854.1	100%	0.0
	WP_011250460.1 Cbf5	32%	$4x10^{-4}$
Cbf5 (TK1509) BAD85698	WP_011250460.1 Cbf5	100%	0.0
	WP_011251072.1 Nol1/Nop2	40%	$1x10^{-8}$
Nop10 (TK1101) BAD85290.1	WP_011250052.1 Nop10	100%	0.0
	WP_011250896.1 IF-2gamma	63%	$2x10^{-6}$
Gar1 (TK2286) BAD86475	WP_011251236.1 Gar1	100%	0.0
L7Ae (TK1311) BAD85500	WP_048053845.1 L7Ae	100%	0.0

Table S3. Oligonucleotides used during this work for the disruption of selected ORFs in the genome of *T. kodakarensis* and for analysis of gene expression.

* Oligonucleotides are indicated with black arrows in Fig. 1A. RT-PCR amplifications with these pairs of primers are shown in Fig. 1C.

use	primer name	sequence 5' to 3'
**Construction of Δ*cbf5* null mutant strain**		
amplification of target TK1509 gene with ~1 kb flanking sequences	CBF5-F	CTGTCTAGAGCTGCTGACCTTTCCGACACG
	CBF5-R	CAGCCGCGGCCCTGTCGAGCCTGCCGAAGG
primers used for inverse PCR	CBF5-InvF	ATGGAGGGGAGGAAGGGCCTCAAGCTGATA
	CBF5-InvR	CACCTACAGATCTTTAAGAGCCCCAACAAA
**Construction of Δ*pus10* KO mutant strain**		
amplification of target gene TK0903 with ~1 kb flanking sequences	PUS10-F	GCTCTAGAGGCTATCCGCTACCGCTGAGAATTGC
	PUS10-R	GCGGATCCGCTGGCAATGTCAATGTATCC
primers used for inverse PCR	PUS10-InvF	GTGGTGAAAAATTTATAAACGCTTCACCAG
	PUS10-InvR	CTGGCCTTCTCGACTATCATGCTCCCACCC
**Construction of Δ*nop10* KO mutant strain**		
amplification of target gene TK1101 with ~1 kb flanking sequences	NOP10-F	GACTCTAGACCCGGCCACTCCCGTCAGGT
	NOP10-R	GCGGATCCAACAGCCAGTATCGTTATCGC
primers used for inverse PCR	NOP10-InvF	TGAAGGAAACGATGATTTACCTGCTTGAGAGG
	NOP10-InvR	TCAGGAACCCCTCTTCTTAACTTTCTTAATCC
**Construction of Δ*gar1* KO mutant strain**		
amplification of target gene TK2286 with ~1 kb flanking sequences	GAR1-F	TCACCATGGAAAAATCAACGCTCCTGTTC
	GAR1-R	GTAGGATCCGAGAATAAGCTCTGGCTGGA
primers used for inverse PCR	GAR1-InvF	GGGTGAGAGGGATTAGCCCGAAGAGGGTATG
	GAR1-InvR	TTCATAGCCCCACCCCAAAACCAGGTTTCC
**Test for the effect of Δ*cbf5* KO on adjacent gene expression**		
amplication of adjacent ORF TK1508*	TK1508-F	ATG GAG GGG AGG AAG GGC CTC AAG CTG ATA
	TK1508-R	CTA AAA TCC CCG TAT TAT GTC CCA CAC AAC
**Test for the effect of Δ*pus10* KO on adjacent gene expression**		
amplication of adjacent ORFs TK0901-0902*	TK0901-0902-F	ATG GTT AAG AAG GCG CAC AGC TTC AGG AGG
	TK0901-0902-R	TCA CTC GAG GGG CCG GTA TTC GTC AAC TAA
**Test for the effect of Δ*nop10* KO on adjacent gene expression**		
amplication of adjacent ORF TK1102*	TK1102-F	ATG AAG GAA ACG ATG ATT TAC CTG CTT GAG
	TK1102-R	TCA GAG GTA GCC CCT GTC CTC CTC TTC CTG
**Test for the effect Δ*gar1* KO on adjacent gene expression**		
amplication of adjacent ORF TK2287*	TK2287-F	ATT AGC CCG AAG AGG GTA TGC CCT ATT TGC
	TK2287-R	TCA TAT CGG GAC GTT TAT GCC GAG CTT TTC
